# Editorial: Revolutionizing nutritional epidemiology: harnessing digital health, AI, and big data for population-level disease prevention and management

**DOI:** 10.3389/fnut.2026.1910148

**Published:** 2026-08-18

**Authors:** Leandro Oliveira, Marta Esgalhado

**Affiliations:** CBIOS (Research Center for Biosciences and Health Technologies), ECTS (School of Health Sciences and Technologies), Lusófona University, Lisbon, Portugal

**Keywords:** artificial intelligence, big data, digital health, nutritional epidemiology, population-level disease prevention

Nutritional epidemiology is being transformed by digital health technologies, artificial intelligence (AI), machine learning (ML), large language models (LLMs), and big data analytics. Traditionally, the field has relied on dietary assessment methods, including food frequency questionnaires, food records, and 24-h recalls, to investigate diet-health relationships. Despite their important contribution to nutrition research and public health, these methods remain limited by recall bias, measurement error, respondent burden, and their inability to capture the dynamic nature of dietary behaviors (1).

**Figure 1:**
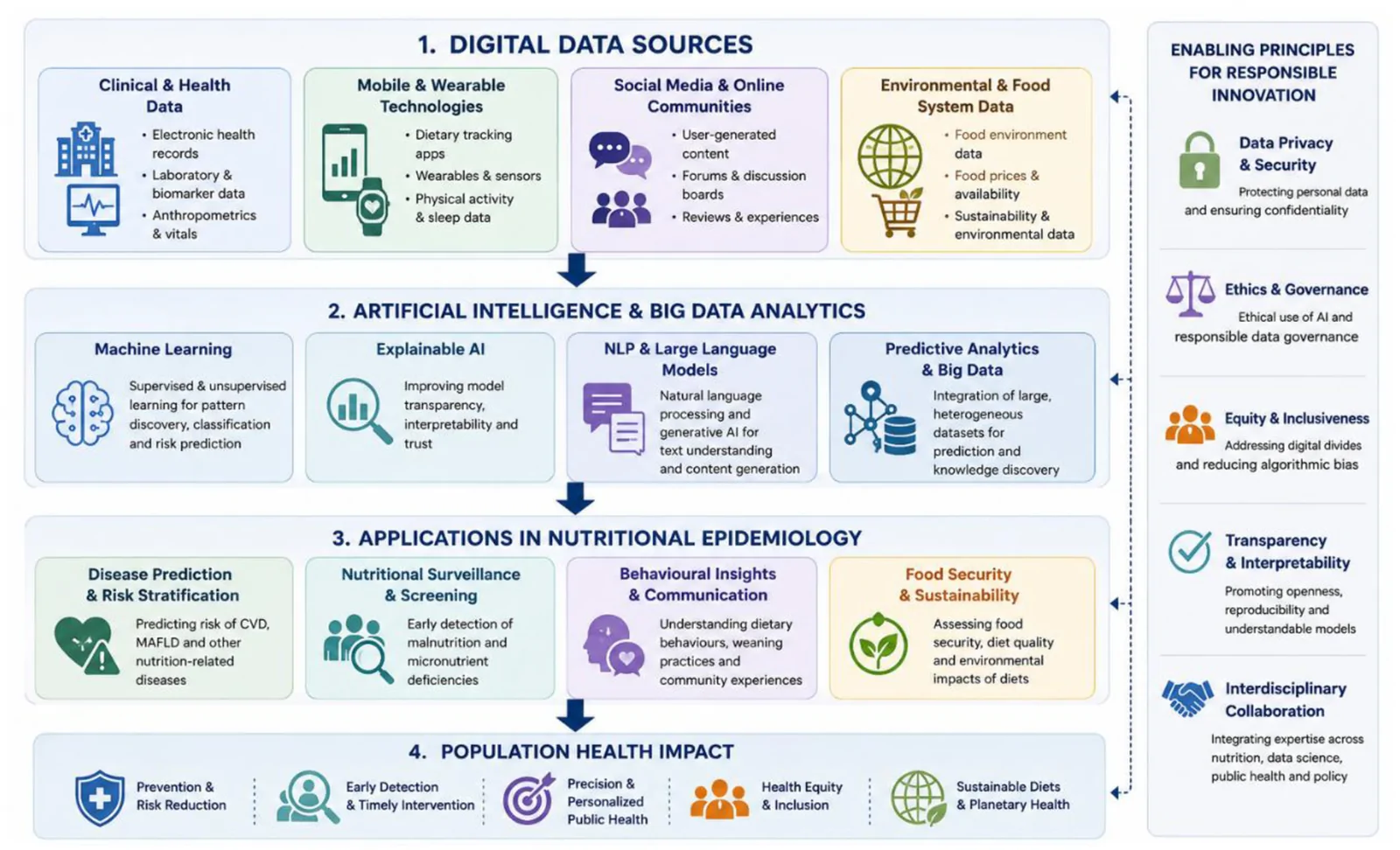
The digital transformation of nutritional epidemiology. This image was generated using ChatGPT (OpenAI, San Francisco, CA, USA), using the GPT-5.6 model.

The rapid expansion of digital technologies offers unprecedented opportunities to overcome many of these limitations. Mobile health applications, wearable sensors, electronic health records, social media platforms, environmental databases, and AI-powered analytical tools are generating vast quantities of health-related information. These data sources provide new possibilities for real-time monitoring, large-scale behavioral assessment, precision nutrition, and population-level disease prevention (2, 3). As illustrated in Figure 1, nutritional epidemiology is increasingly evolving toward an integrated ecosystem in which diverse digital data sources are transformed into actionable public health knowledge through AI-driven analytical approaches. The figure synthesizes the conceptual framework emerging from the studies included in this Research Topic, illustrating how heterogeneous digital data sources are integrated through AI and big data analytics to support disease prediction, nutritional surveillance, behavioral research, food security assessment, and sustainability. It also emphasizes that responsible implementation depends on data quality, transparency, ethical governance, and interdisciplinary collaboration. Rather than representing isolated technological advances, the framework highlights how these interconnected components collectively support the transition toward precision nutrition and data-driven public health. The 11 articles assembled in this Topic provide a timely and comprehensive overview of this transformation. Collectively, they demonstrate how emerging digital technologies are reshaping nutritional epidemiology by enabling innovative approaches to disease prediction, nutritional surveillance, behavioral analysis, food security assessment, and sustainability research. Importantly, these studies also highlight both the opportunities and methodological challenges associated with the integration of AI and big data into nutrition and public health research.

One of the most prominent themes emerging from this Research Topic is the growing use of machine learning for disease prediction and risk stratification. *Do nutritional variables improve cardiovascular disease prediction? A comparative machine learning analysis* examined the contribution of nutritional variables to cardiovascular risk prediction (Toprak et al.), while *Combined analysis of the triglyceride-glucose index and melanin-concentrating hormone in metabolic dysfunction-associated fatty liver disease: a machine learning-based study* applied machine learning to improve the identification of metabolic dysfunction-associated fatty liver disease (Hong et al.). Similarly, *Relationship between iodine nutritional status and low handgrip strength in a Northwestern Chinese cohort: construction of a predictive nomogram model* developed an AI-based model for predicting low handgrip strength (Liu et al.), whereas *Trajectories of health conditions predict cardiovascular disease risk among middle-aged and older adults: a national cohort study* demonstrated how longitudinal health trajectories can enhance cardiovascular risk prediction (Li et al.). Collectively, these contributions highlight the potential of multidimensional data and AI-driven approaches to support precision public health and personalized prevention strategies.

These contributions also provide important insights regarding the role of nutritional variables within predictive frameworks. While dietary factors are widely recognized as major determinants of chronic disease risk, evidence regarding their incremental contribution to predictive models remains mixed. The study entitled *Do nutritional variables improve cardiovascular disease prediction? A comparative machine learning analysis* demonstrated that the inclusion of detailed nutritional variables produced only modest and model-dependent improvements in predictive performance (Toprak et al.). These findings reinforce an important principle in modern data science: increasing the number of variables does not necessarily improve predictive accuracy, while model interpretability and robustness remain equally important (4).

Another major contribution of this Research Topic concerns the use of digital health technologies for nutritional surveillance and public health monitoring. Traditional nutritional surveillance systems often require substantial financial and human resources and may provide information only at infrequent intervals. Digital tools offer opportunities to complement these systems by enabling continuous and scalable data collection. *D2A: a community-led smartphone tool for malnutrition screening in Kenya* demonstrates how mobile technologies can facilitate community-led nutritional surveillance and early identification of malnutrition, particularly in resource-limited settings (Bhavnani and Link). Beyond clinical prediction and surveillance, this Research Topic highlights the increasing value of user-generated digital data in nutritional epidemiology. Social media platforms, online forums, and virtual communities generate extensive information regarding dietary practices, feeding behaviors, perceptions, and lived experiences. Studies analyzing discussions on breastfeeding, breast pumping, and weaning using natural language processing and large language models illustrate the growing potential of user-generated digital data to identify behavioral patterns, psychosocial concerns, and unmet informational needs. The results highlighted psychosocial concerns, behavioral determinants, and informational needs that would be difficult to capture through traditional survey-based approaches.

The use of social media and digital trace data represents an important methodological innovation within nutritional epidemiology. Unlike conventional data collection methods, which often depend on structured questionnaires administered at specific time points, digital platforms provide access to naturally occurring discussions that reflect real-world experiences and decision-making processes. Consequently, these approaches may contribute to a more comprehensive understanding of nutrition-related behaviors while complementing established epidemiological methods (5).

Large language models are emerging as another important area of innovation. Recent advances in generative AI have demonstrated remarkable capabilities in information retrieval, text synthesis, and decision support (6, 7). Among the articles included in this Topic, *Dietary guidance for pregnant women using DeepSeek-R1 and ChatGPT-4.0: a comparative analysis* provides valuable insights into the strengths and current limitations of large language models for nutrition guidance during pregnancy (Gao et al.). Although AI-generated recommendations may support nutrition education and personalized guidance, concerns regarding accuracy, reliability, transparency, and bias remain highly relevant. Continued evaluation and expert oversight will be essential to ensure that AI-based nutrition advice supports evidence-based practice rather than contributing to misinformation.

The articles also demonstrate how AI and big data approaches can address broader food-system challenges. Food security, food equity, sustainability, and environmental impacts are increasingly recognized as central components of modern nutritional epidemiology. The study entitled *Advancing food equity through explainable AI: identifying place-based factors and conditions of food security* illustrates how explainable AI can improve transparency while addressing complex determinants of food security (Hoglund and Park). Similarly, *Dietary assessment at the confluence of public and planetary health: introduction of the DIEM (Dietary Impacts on Environmental Measures) scoring system* introduced an integrated framework combining dietary quality and environmental indicators, reflecting the growing recognition that nutrition and sustainability should be considered together rather than as separate domains (Katz et al.). These developments align closely with emerging concepts of sustainable diets and planetary health, which emphasize the interconnected nature of human and environmental wellbeing (8).

While the studies included in this Research Topic collectively demonstrate the transformative potential of AI and digital health technologies, they also highlight that the field remains at an early stage of development. Despite the considerable progress reported across the included studies, important knowledge gaps remain regarding the generalizability of AI models across diverse populations, the integration of heterogeneous digital nutrition data, and the translation of AI-driven innovations into routine clinical and public health practice. Future research should prioritize prospective validation of predictive models across diverse populations, harmonization of digital nutrition data sources, standardized reporting of AI methodologies, explainable AI, and integration of multimodal data from clinical, behavioral, environmental, and digital sources. These priorities will be essential for translating methodological advances into robust public health and clinical applications. Despite the considerable promise of these innovations, important challenges remain. The increasing use of digital data raises concerns regarding privacy, confidentiality, algorithmic bias, reproducibility, and ethical governance. In addition, external validation across independent populations, standardized reporting of AI methodologies, data quality, missing data handling, model calibration, and evolving regulatory frameworks will be fundamental before these technologies can be widely implemented in clinical and public health settings. Digital inequalities may result in underrepresentation of vulnerable populations within datasets used to train predictive models, potentially exacerbating existing health disparities. Furthermore, the complexity of many AI systems can limit interpretability and create barriers to implementation within clinical and public health settings. Addressing these challenges will require multidisciplinary collaboration among nutrition scientists, epidemiologists, data scientists, healthcare professionals, policymakers, and ethicists (9, 10).

In conclusion, the articles brought together in this Topic provide compelling evidence that digital health technologies, artificial intelligence, large language models, and big data analytics are reshaping nutritional epidemiology. From disease prediction and nutritional surveillance to behavioral analysis, food security assessment, and sustainability research, these innovations offer powerful opportunities to strengthen evidence generation and public health action. As the field continues to evolve, maintaining scientific rigor, transparency, ethical governance, and public health relevance will be essential to fully realize the potential of these emerging tools for disease prevention and health promotion. Ultimately, the future of nutritional epidemiology will depend not only on technological innovation but also on ensuring that digital tools remain scientifically rigorous, transparent, equitable, reproducible, and capable of generating meaningful improvements in population health.
